# A Rare Glimpse of Paleoarchean Life: Geobiology of an Exceptionally Preserved Microbial Mat Facies from the 3.4 Ga Strelley Pool Formation, Western Australia

**DOI:** 10.1371/journal.pone.0147629

**Published:** 2016-01-25

**Authors:** Jan-Peter Duda, Martin J. Van Kranendonk, Volker Thiel, Danny Ionescu, Harald Strauss, Nadine Schäfer, Joachim Reitner

**Affiliations:** 1 Department of Geobiology, Geoscience Centre, Georg-August-University Göttingen, Goldschmidtstr. 3, 37077, Göttingen, Germany; 2 ‘Origin of Life’ Group, Göttingen Academy of Sciences and Humanities, Theaterstraße 7, 37073, Göttingen, Germany; 3 Australian Centre for Astrobiology and School of Biological, Earth and Environmental Sciences, University of New South Wales, Kensington, NSW 2052, Australia; 4 Department of Experimental Limnology, Leibniz Institute for Freshwater Ecology and Inland Fisheries (IGB), Alte Fischerhütte 2, 16775, Stechlin, Germany; 5 Institut für Geologie und Paläontologie, Westfälische Wilhelms-Universität Münster, Corrensstraße 24, 48149, Münster, Germany; The University of Akron, UNITED STATES

## Abstract

Paleoarchean rocks from the Pilbara Craton of Western Australia provide a variety of clues to the existence of early life on Earth, such as stromatolites, putative microfossils and geochemical signatures of microbial activity. However, some of these features have also been explained by non-biological processes. Further lines of evidence are therefore required to convincingly argue for the presence of microbial life. Here we describe a new type of microbial mat facies from the 3.4 Ga Strelley Pool Formation, which directly overlies well known stromatolitic carbonates from the same formation. This microbial mat facies consists of laminated, very fine-grained black cherts with discontinuous white quartz layers and lenses, and contains small domical stromatolites and wind-blown crescentic ripples. Light- and cathodoluminescence microscopy, Raman spectroscopy, and time of flight—secondary ion mass spectrometry (ToF-SIMS) reveal a spatial association of carbonates, organic material, and highly abundant framboidal pyrite within the black cherts. Nano secondary ion mass spectrometry (NanoSIMS) confirmed the presence of distinct spheroidal carbonate bodies up to several tens of μm that are surrounded by organic material and pyrite. These aggregates are interpreted as biogenic. Comparison with Phanerozoic analogues indicates that the facies represents microbial mats formed in a shallow marine environment. Carbonate precipitation and silicification by hydrothermal fluids occurred during sedimentation and earliest diagenesis. The deciphered environment, as well as the δ^13^C signature of bulk organic matter (-35.3‰), are in accord with the presence of photoautotrophs. At the same time, highly abundant framboidal pyrite exhibits a sulfur isotopic signature (δ^34^S = +3.05‰; Δ^33^S = 0.268‰; and Δ^36^S = -0.282‰) that is consistent with microbial sulfate reduction. Taken together, our results strongly support a microbial mat origin of the black chert facies, thus providing another line of evidence for life in the 3.4 Ga Strelley Pool Formation.

## Introduction

Detecting early life on Earth is challenging, as many potential biosignatures may also be explained by abiotic processes. It has therefore been suggested that potential biosignatures in Paleoarchean rocks should only be considered valid if all possible pathways of abiological formation are ruled out (i.e. the “null hypothesis”; [1]–[4]). However, this approach is problematic, as single rock characteristics can commonly be explained by several processes, making the identification of any unambiguous traces of early life on Earth almost impossible. Nevertheless, it may be possible to identify life in Paleoarchean strata with a higher degree of probability through convergent lines of evidence obtained by combined geobiological approaches.

Paleoarchean rocks from the Pilbara Craton (Western Australia) exhibit a variety of evidence for early life on Earth over the period of 3.5–3.0 Ga (see [5]–[16], and references therein). Important traces of microbial life include widespread stromatolites (i.e. laminated benthic microbial deposits; [17]) and microfossils in the c. 3.4 Ga Strelley Pool Formation of the East Pilbara Terrane (e.g., [5], [7]-[9], [18]-[24]). However, some authors have argued for an abiotic origin of the stromatolites [25], [26]. Similarly, the biogenicity of stromatolites from the 3.48 Ga Dresser Formation has also been debated (e.g., [7], [25], [27], [28]). This debate is mainly due to the fact that some stromatolite-like structures could theoretically be formed by abiotic processes, such as the deposition of mineral crusts [1], [29]–[33]. For example, it has been shown that abiotically formed stromatolite-like structures in natural environments (e.g., geyserites; [29]) and in laboratory experiments (e.g., from the precipitation of synthetic colloids; [33]) can exhibit complex characteristics such as columnar and branched growth forms, non-isopachous laminae and wrinkle structures that are commonly regarded as hallmarks of biogenicity.

Evaporitic precipitation of mineral crusts [25] and direct precipitation from hydrothermal solutions [26] have been proposed as abiotic pathways for the formation of the Strelley Pool stromatolites. However, detailed observations of sedimentary facies and fabrics, particularly the association of sedimentary features and layering relationships, generally point to a biogenic origin for most, if not all of the stromatolites (e.g., [7], [8], [19], [34], [35]). The biogenicity of the Strelley Pool stromatolites is further supported by trace element signatures of carbonates and cherts, which point to precipitation from seawater [35], [36]. In addition, nanoscale secondary ion mass spectrometric analysis (NanoSIMS) has revealed C, N and S distributions in organic material that are in good agreement with a biological origin [12]. Finally, the discovery of microfossils from the Strelley Pool Formation confirms biological activity during sediment accumulation [9]-[11].

Here we describe a new type of microbial mat facies within the Strelley Pool Formation, which consists of black cherts and contains small domical stromatolites as well as discontinuous white quartz layers and lenses (e.g., [6], [7], [24], [37]). By combining detailed field and petrographic observations with biogeochemical data (isotope distribution patterns, stable carbon isotopes of bulk organic matter, multiple sulfur isotopes of pyrite crystals), we draw conclusions on the paleoenvironment, geomicrobiology, and taphonomical processes. The results confirm the existence of microbial mat systems in the 3.4 Ga Strelley Pool Formation and extend our knowledge about habitats in which early life thrived.

## Geological Framework

**Fig 1:**
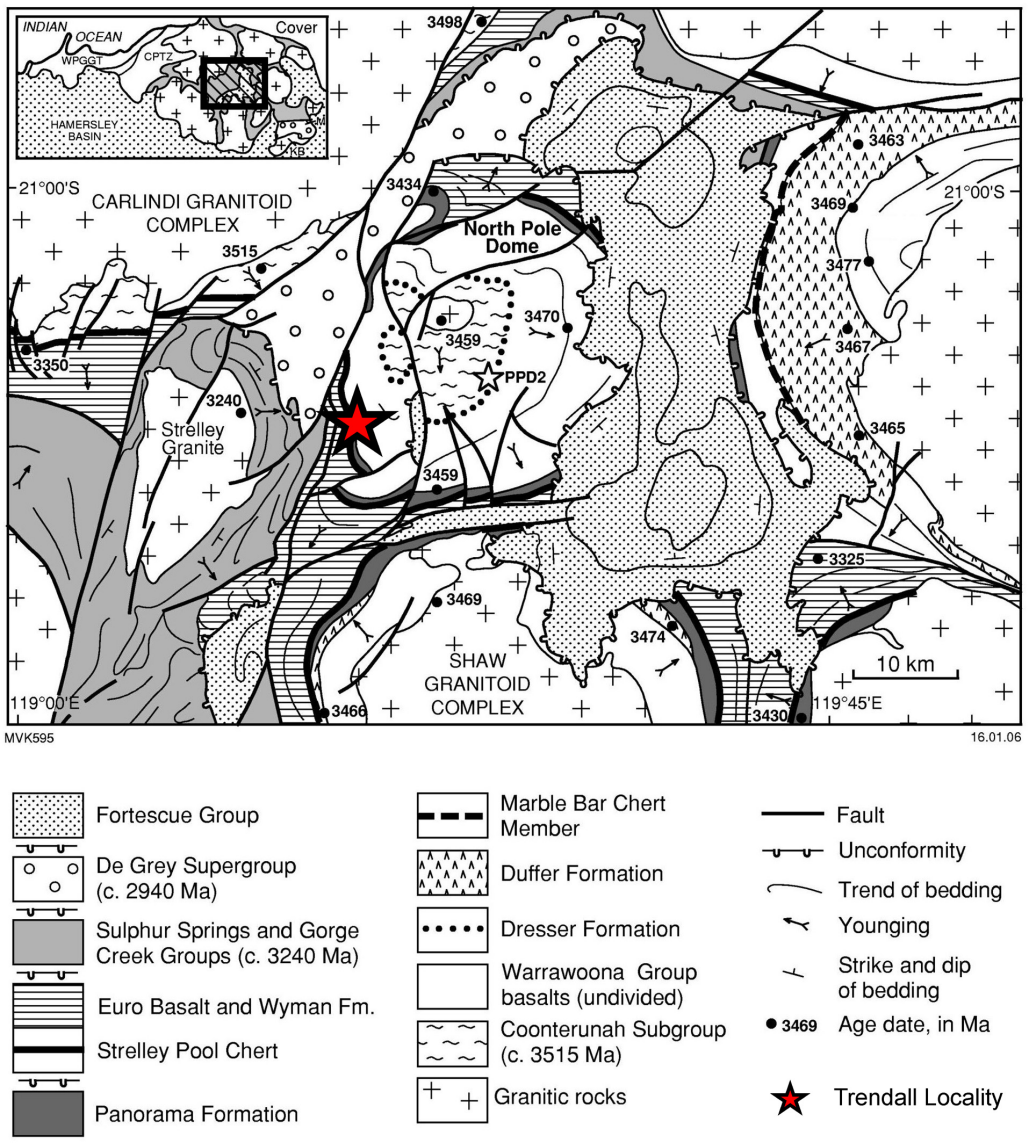
Study area (modified after [90], with permission from the copyright holder, the Geological Survey of Western Australia). The analyzed black chert facies crops out at the Trendall locality in the northern part of Western Australia.

The Trendall locality of the Strelley Pool Formation [7], [19] is located in the northern part of Western Australia (Fig 1). Geologically, this site belongs to the Panorama Greenstone Belt, one of several greenstone belts of the East Pilbara Terrane (e.g., [38]). The Trendall locality is situated in the southwestern part of the North Pole Dome, which is cored by the North Pole Monzogranite (3459 ± 18 Ma; [39]).

**Fig 2:**
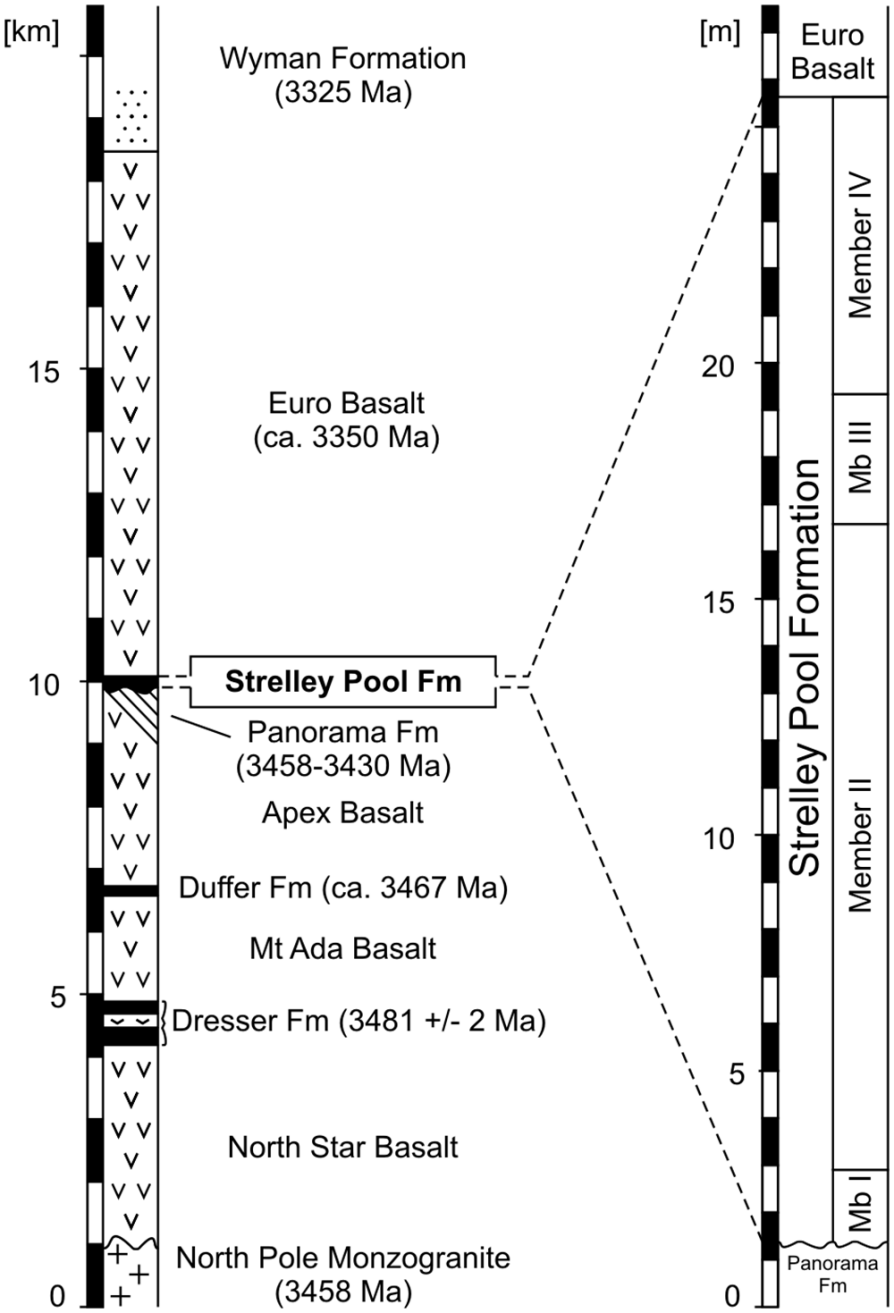
Stratigraphical context of the Strelley Pool Formation (representative sections; modified after [8], [35], [36], [43], [90]). The black chert facies corresponds to Member III.

The 3.43–3.35 Ga Strelley Pool Formation (Fig 2: previously known as the Strelley Pool Chert (e.g., [18]), but renamed by [23]) overlies rocks of the 3.53–3.43 Ga Warrawoona Group on an erosional unconformity that extends over the entire East Pilbara Terrane [5], [20], [38], [40], [41]. At the Trendall locality, the Strelley Pool Formation unconformably overlies the Mount Ada Basalt (3469 ± 3 Ma; [42]) and is (para-) conformably overlain by the Euro Basalt (3350 ± 3 Ma; [43]). Detailed information on the local stratigraphy at the Trendall locality is provided in [6] and [21].

The Strelley Pool Formation consists of largely silicified sedimentary and volcaniclastic rocks and is usually less than 30 m thick [7], [23]. The formation can be subdivided into several members, with the exact number depending on the locality (see [23]). The Trendall locality consists of four members, from base to top: (I) chert pebble to boulder conglomerates and quartz-rich sandstones; (II) laminated and stromatolitic carbonates with common pseudomorphed evaporitic crystal splays; (III) layered black and white cherts with small, well-laminated, iron-rich domical stromatolites; and (IV) an unconformably overlying sequence of polymict conglomerates, sandstones, and volcanic ash [7], [8], [21], [22]. These members represent a rocky shoreline and tide-dominated beach environment (Member I), an isolated peritidal stromatolite/evaporite platform (Member II), a mixed shallow marine/hydrothermal environment (Member III), and a distal deep water environment influenced by hydrothermal processes (Member IV) [7], [8], [11], [21], [22]. This study provides a detailed examination of the black cherts from Member III.

## Methods

Samples were collected at the Trendall locality and the corresponding section on the other side of the Shaw River (21°12'24.76"S; 119°18'27.70"E) (Fig 1) in 1997–98, prior to the site becoming a geological reserve. Permits were obtained from the Geological Survey of Western Australia for subsequent field work in this area, during 2014, when outcrops were inspected but not sampled. The field studies did not involve endangered or protected species.

Petrographic analysis was conducted with a Zeiss SteREO Discovery.V8 stereomicroscope (transmitted- and reflected light) linked to an AxioCam MRc 5-megapixel camera. For cathodoluminescence (CL) microscopy a Citl CCL 8200 Mk3A cold-cathode system (operating voltage of c.15 kV; electric current of c. 250–300 μA) linked to a Zeiss Axiolab microscope and cooled SPOT-CCD camera was used. Field emission scanning electron microscopy (Fe-SEM) was performed using a Carl Zeiss LEO 1530 Gemini system.

Raman spectra were recorded using a Horiba Jobin Yvon LabRam-HR 800 UV spectrometer (focal length of 800 mm) attached to an Olympus BX41 microscope. For excitation an Argon ion laser (Melles Griot IMA 106020B0S) with a laser power of 20 mW was used. The laser beam was focused onto the sample using an Olympus MPlane 100 x objective with a numerical aperture of 0.9. The laser beam was dispersed by a 600 l/mm grating on a CCD detector with 1024 x 256 pixels, yielding a spectral resolution of <2 cm^-1^ per pixel. Data were acquired over 30 s for a spectral range of 100–2000 cm^-1^ or 100–4000 cm^-1^. The spectrometer was calibrated by using a silicon standard with a major peak at 520.4 cm^-1^. All spectra were recorded and processed using the LabSpecTM database (version 5.19.17; Jobin-Yvon, Villeneuve d’Ascq, France).

Stable carbon isotope measurements (^12^C, ^13^C) on bulk organic matter were conducted at the Centre for Stable Isotope Research and Analysis at the University of Göttingen (KOSI), Germany, using a CN/IRMS (NA-2500 CE-Instruments/Finnigan MAT Delta plus). C. 100 mg of powdered whole rock material were analyzed. Data are expressed as delta value relative to Vienna Pee Dee Belemnite (V-PDB). For internal calibration an acetanilide standard was used. δ^13^C values reported here have an analytical error of <0.2‰.

Multiple sulfur isotope measurements (^32^S, ^33^S, ^34^S, and ^36^S) were performed at the Institut für Geologie und Paläontologie (University of Münster, Germany). Pyrite sulfur was liberated from sample powder via acidic chromous (II) chloride reduction (cf. [44]). Resulting silver sulfide precipitates were subsequently converted to sulfur hexafluoride (SF_6_) via fluorination in nickel tubes with a fivefold excess of F_2_ for at least 8 hours at 300°C (cf. [45]). The SF_6_ was cryogenically and chromatographically purified before being introduced into a ThermoFinnigan MAT 253 via a dual-inlet system. Results are expressed as delta value relative to the Vienna Canyon Diablo Troilite (V-CDT). Δ^33^S and Δ^36^S values were calculated from δ^33^S, δ^34^S, and δ^36^S following [46], [47]. The analytical error was <0.3‰ for δ^34^S, <0.01‰ for Δ^33^S and <0.1‰ for Δ^36^S. Accuracy of the multiple sulfur isotope measurements was determined by replicate analyses of the international reference material IAEA-S1 (δ^34^S = -0.30‰).

Samples for nano secondary ion mass spectrometry (NanoSIMS) were cut using a carefully pre-cleaned precision saw with a diamond-studded blade, polished, extensively rinsed with pre-cleaned H_2_O and organic solvents, and dried under a stream of Ar. NanoSIMS analyses were conducted with a Cameca NanoSIMS 50/50L based at the Max-Planck-Institute for Marine Microbiology in Bremen, Germany. Analyzed ions included ^12^C^-^, ^13^C^-^, ^12^C^14^N^-^, ^12^C^15^N^-^, ^32^S^-^, and ^34^S^-^. The mass resolution during all measurements was >8000. For the analysis the sample was pre-sputtered with a 16 keV, 1.1–3.5 pA CsC primary ion beam focused on a spot of c. 120 nm in diameter that was stepped over the sample in a 256 x 256 pixel raster with a counting time of 1 ms per pixel. For each region of interest 30 to 100 planes were acquired at a raster size of 30 μm x 30 μm. The data were analyzed using the Look@NanoSIMS software [48].

Time of flight—secondary ion mass spectrometry (ToF-SIMS) images and spectra were recorded from a chert section cut with a carefully pre-cleaned Buehler precision saw. The surface was extensively rinsed with pre-cleaned H_2_O and organic solvents, and dried under a stream of Ar. The sample was stored under Ar in a glass container for transport. Analysis was performed at SP Technical Research Institute of Sweden (Borås) using a ToF-SIMS IV instrument (ION-TOF GmbH, Münster, Germany). The analyzed area was 300 μm × 300 μm and was scanned using 25 keV Bi_3_^+^ primary ions at a pixel resolution of c. 2.3 μm. Low energy electron flooding was used for charge compensation. Spectra were obtained at a mass resolution of m/Δm = 5000. Peak assignments were based on the exact mass and comparison with the theoretical isotope distributions of the ions of interest.

## Results

**Fig 3:**
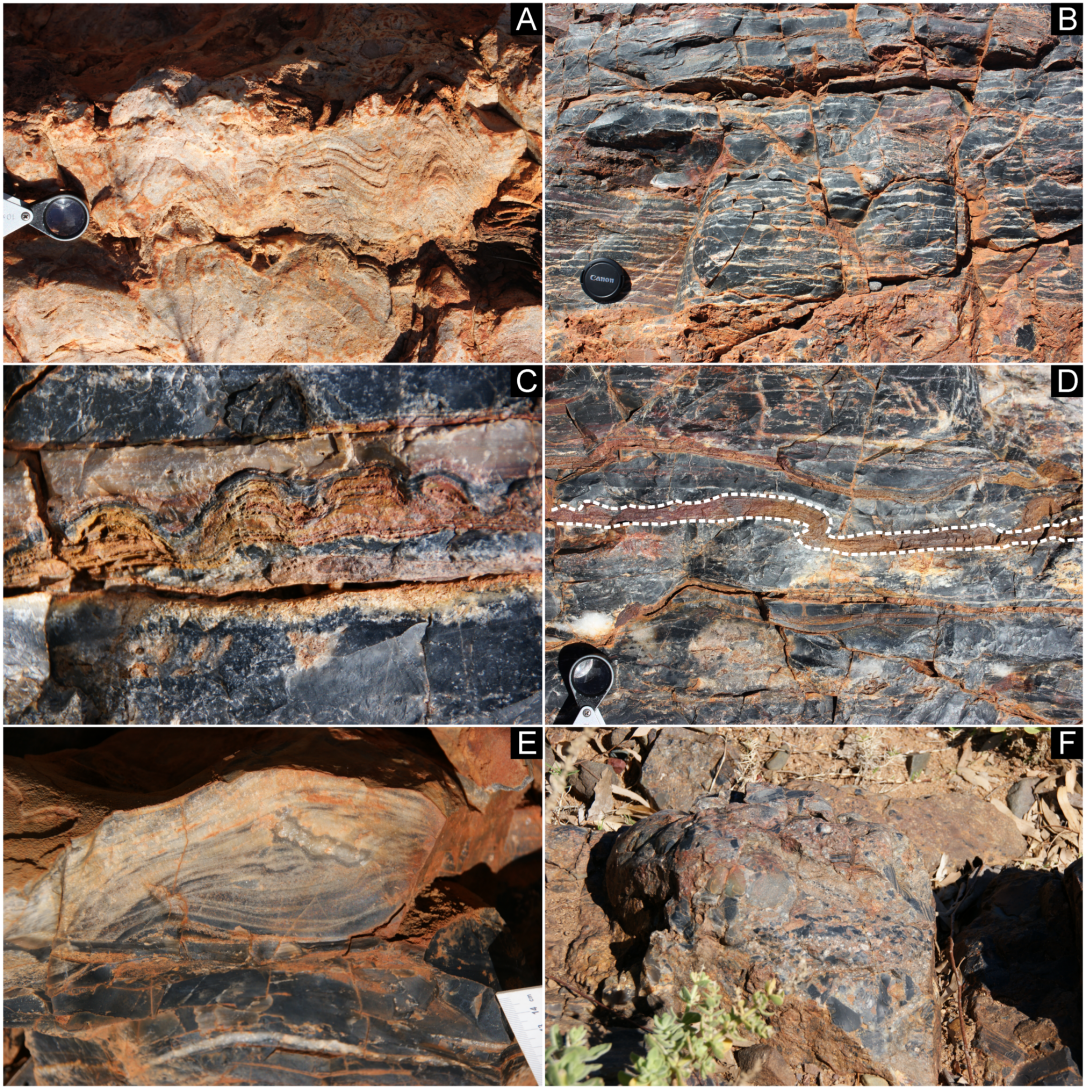
Field observations of the black chert facies. (A) Laminated and stromatolitic carbonates (Member II of the Strelley Pool Formation) below the black chert facies. (B-E) Characteristics of the black chert facies include fenestral fabrics (B), intercalated cm-high stromatolitic layers (C) that locally show ductile deformation (dashed line in D), as well as small-scaled cm-sized cross lamination (E). (F) Conglomerate (Member IV of the Strelley Pool Formation) above the black chert facies.

The black chert facies of this study (Member III) overlies well-laminated stromatolitic dolomitic carbonates of Member II (Figs 2 and 3A). The 1–2 m thick facies consists of very fine-grained black cherts with mm- to cm-thick, discontinuous white quartz layers and lenses that are typically 1 mm—1 cm thick and 10–20 cm wide (Fig 3B). This facies includes a number of cm-thick layers of thinly laminated, ferruginous domical stromatolites in silicified carbonates (Fig 3C), which locally show ductile deformation (Fig 3D) [7]. An additional feature of the lower part of this facies is cm-scale cross lamination in silicified carbonate sandstones (Fig 3E). The black chert facies is unconformably overlain by cobble to boulder conglomerates of Member IV that include clasts of the black chert facies, in addition to boulder-size clasts of unsilicified carbonates (Fig 3F).

**Fig 4:**
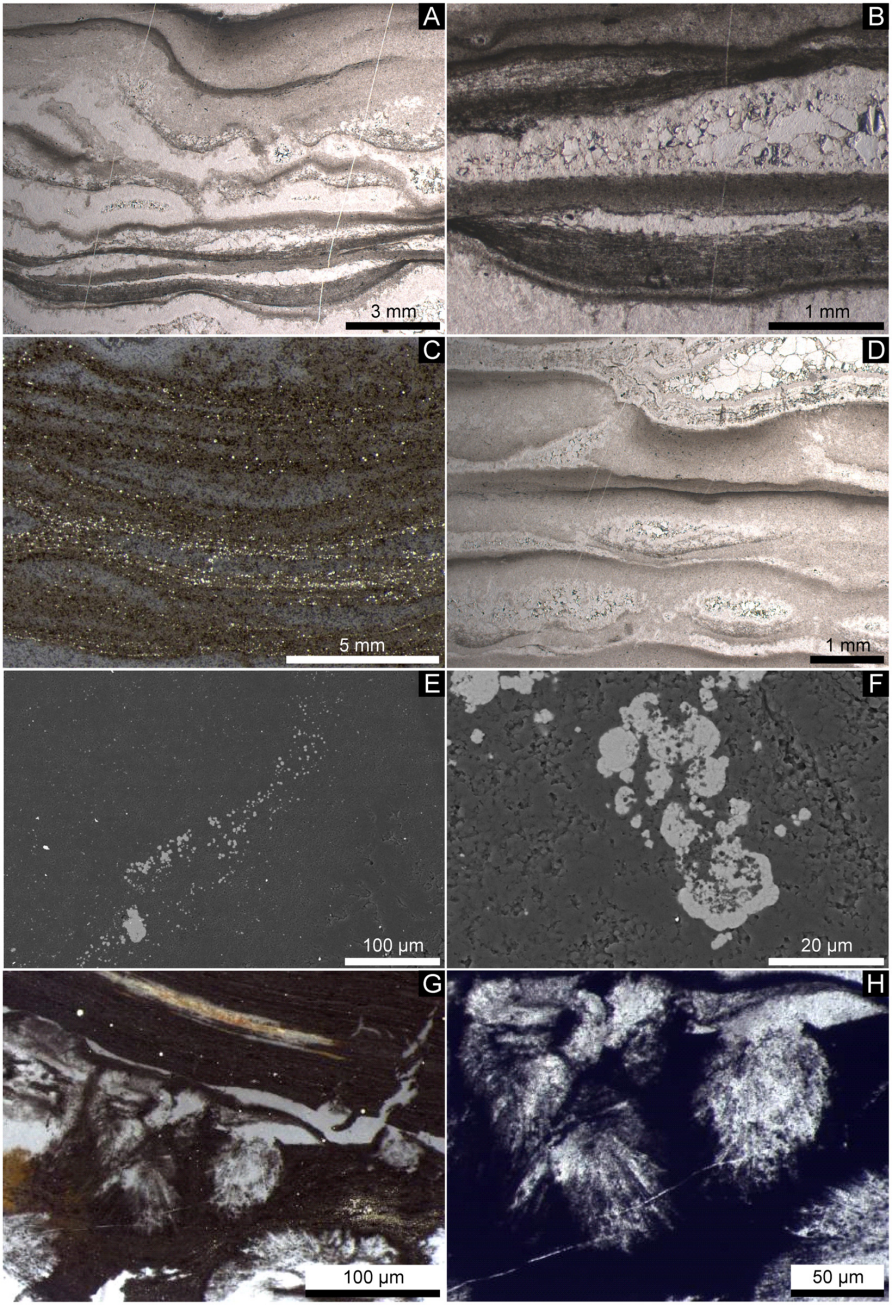
Petrographic observations on the black chert facies. (A-D) Thin sections (A, B, D: transmitted light; C: reflected light). (A) Layers are laterally not continuous in thickness but wavy. (B, C) Dark chert layers consist of a fine grained matrix that is enriched in organic matter and pyrite. Note that the organic material is laterally interwoven (B) and closely associated with pyrite (C). (B, D) Fenestrae are filled by blocky cements. (E, F) SEM photographs of the pyrite crystals. Note that the pyrite crystals are enriched in layers (E) and framboidal in shape (F). (G, H) Clusters of silicified acicular crystals, probably representing chert pseudomorphs after aragonite.

**Fig 5:**
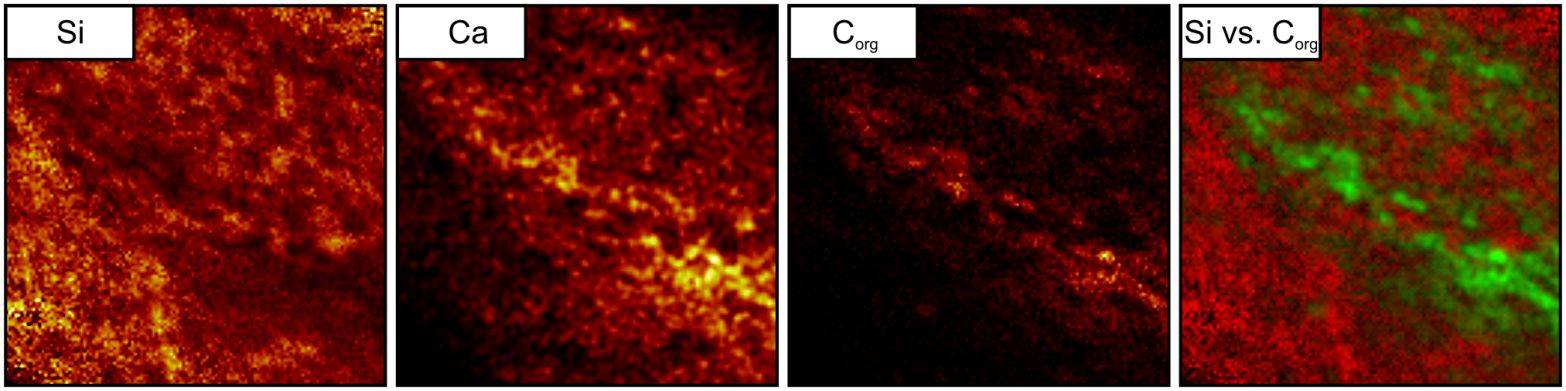
ToF-SIMS ion images of a 300μm × 300μm area of the back chert facies with an organic matter layer in the center. The pixel brightness reflects the signal intensity of (from the left): Si = [Si]^+^, representing the chert matrix; Ca = sum of [Ca]^+^, [CaO]^+^, and [CaOH]^+^, representing CaCO_3_; C_org_ = sum of major hydrocarbon ions [C_2_H_3_]^+^, [C_2_H_5_]^+^, [C_3_H]^+^, [C_3_H_2_]^+^, and [C_3_H_3_]^+^, representing organic matter. Pixel brightness in Ca and color coded overlay of Si (red) and C_org_ (green) is enhanced to increase image contrast.

**Fig 6:**
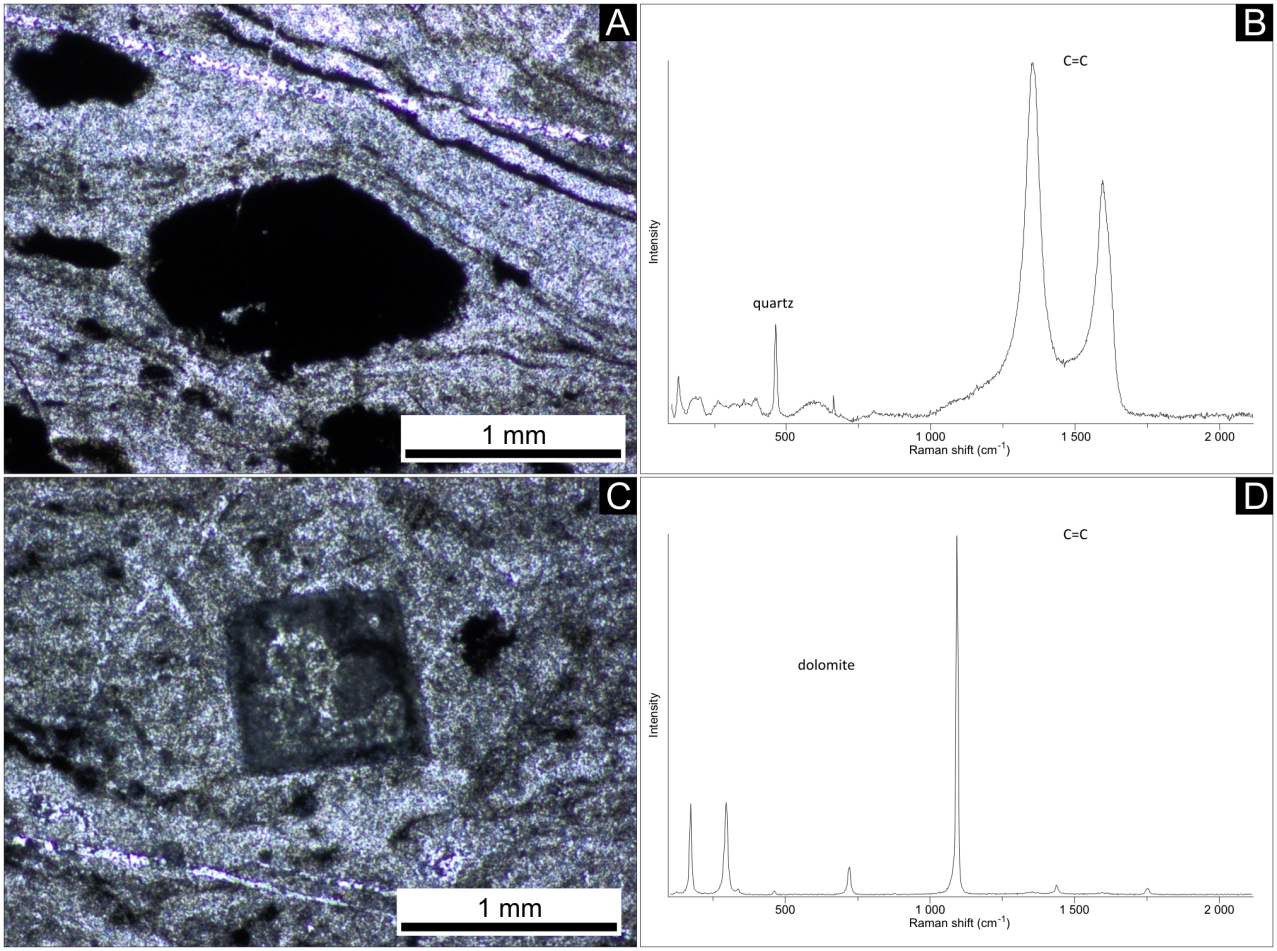
Thin sections (A, C) and corresponding Raman spectra (B, D; point measurements) documenting local occurrences of organic material (A, B) and dolomite (C, D) in some fenestrae.

Lenses and layers of white quartz within the black chert facies vary in thickness and are wavy (Fig 4A). The black chert is enriched in organic matter and fine-grained pyrite (Fig 4B and 4C). The organic material is commonly concentrated in distinct laminae and in depressions directly below quartz lenses and layers (Fig 4B), as confirmed by ToF-SIMS imaging (Fig 5). However, the organic matter also locally appears in the form of organic flakes, as revealed by Raman spectroscopy (Fig 6A and 6B).

**Fig 7:**
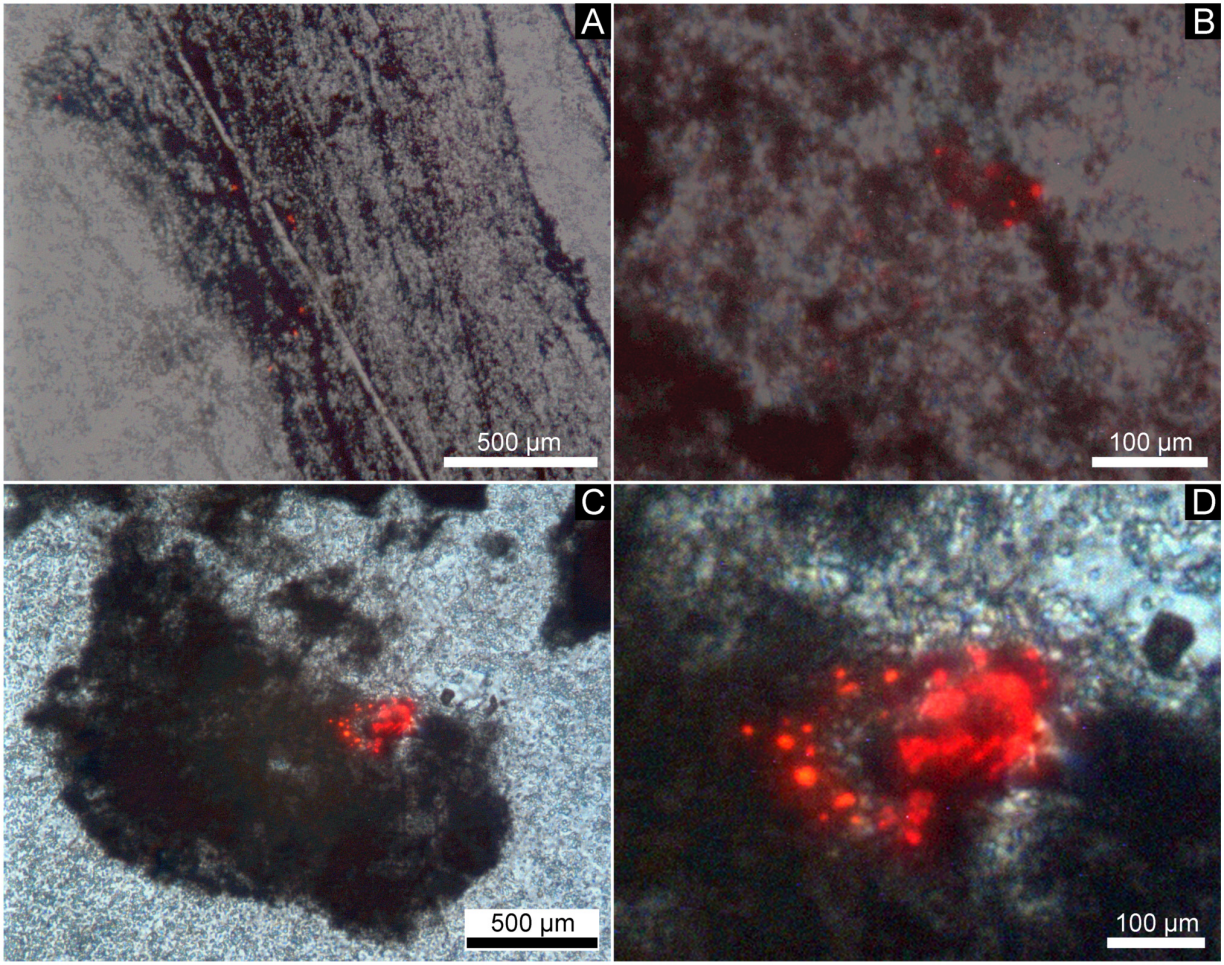
CL overlay photographs of the black chert facies. Note that the calcite aggregates (red luminescence) appear to be linked to organic material (dark colors). (A) Finely laminated layers of organic matter containing CaCO_3_ spheroids (red luminescence). (B) Close up view of (A). (C) Aggregated CaCO_3_ spheroids (red luminescence) associated with organic matter. (D) Close up view of (C).

An unusual feature of the black chert facies is the presence of anhedral calcite aggregates that are typically 0.1–1 mm in size and show a characteristic red CL response induced by intracrystalline Mn^2+^ (e.g., [49], [50]) (Fig 7). Typically, these aggregates are associated with organic matter in distinct layers, as confirmed by ToF-SIMS (Fig 5).

Pyrite is abundant in the black chert layers but absent in the white quartz layers and lenses. Under reflected light, most of the pyrite appears to be associated with enrichments of organic matter (Fig 4C). Single pyrite crystals are typically <20 μm in size (rarely >50 μm). Detailed SEM observations revealed that the pyrite crystals formed in layers, as small aggregates, and have a framboidal texture (*sensu* [51]; Fig 4E and 4F).

The quartz lenses and layers are filled with blocky quartz cement having a typical grain size of <0.5 mm. Larger quartz lenses, >1 mm thick, show multiple layers of isopachous cement at their fringes and coarser blocky cements in their interiors (Fig 4D). Some of the larger lenses contain clusters of very fine, acicular crystal ghosts with pseudo-hexagonal cross-sections (now silicified, but most likely originally aragonite: Fig 4G and 4H) and dolomite rhomboids (mineralogy confirmed by Raman spectroscopy: Fig 6C and 6D).

**Fig 8:**
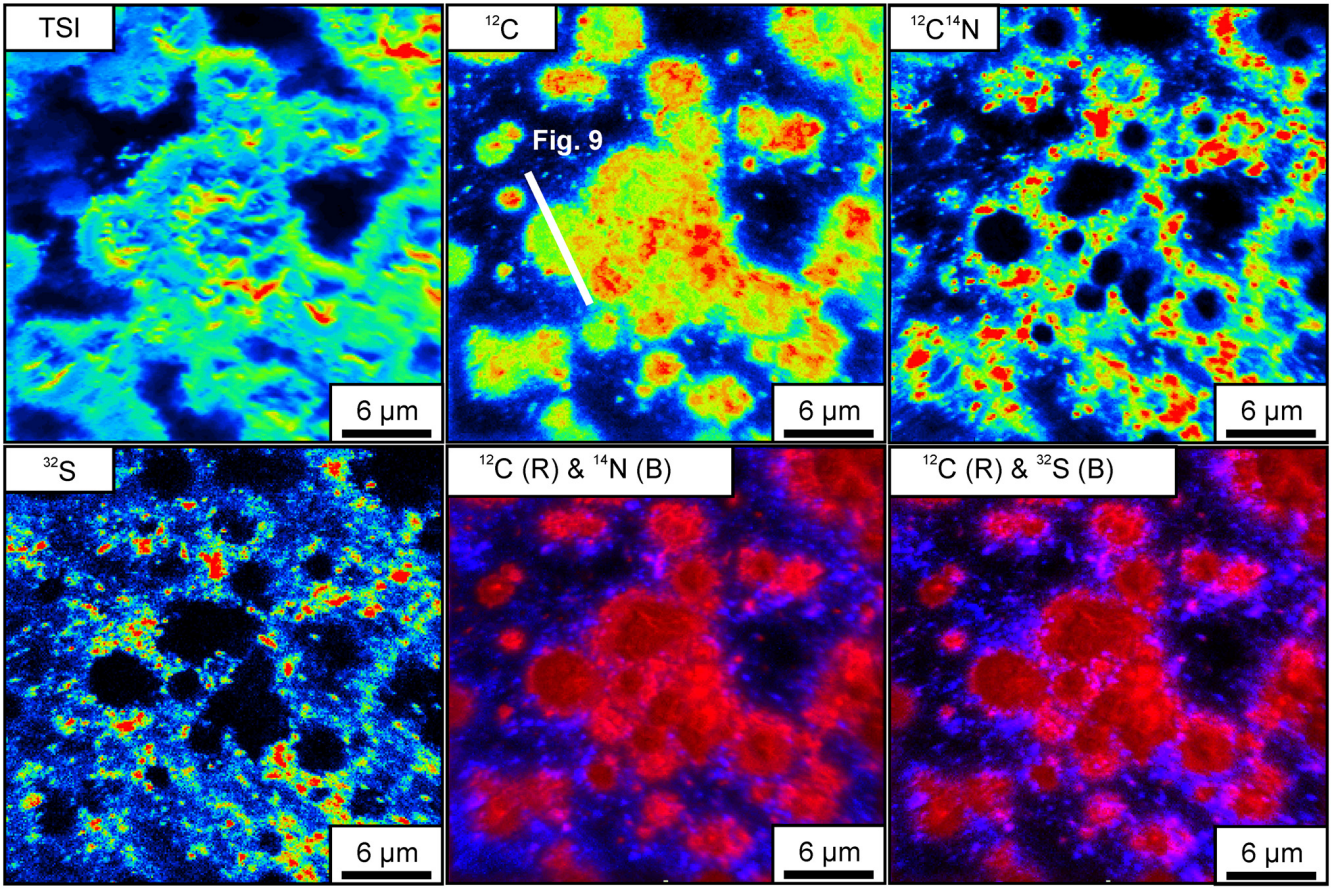
NanoSIMS isotope enrichment maps of organic layers shown in Fig 7A, revealing the presence of spheroids (TSI: Total secondary ions). Note that these bodies are generally enriched in C (^12^C) compared to the surrounding areas. Organic matter (^12^C^14^N) and sulfur (^32^S) are preferentially enriched at the edges, whereas the centers represent carbonate phases. This spatial arrangement of the isotopes is further illustrated in the color-coded overlay maps (^12^C & ^14^N; ^12^C & ^32^S).

**Fig 9:**
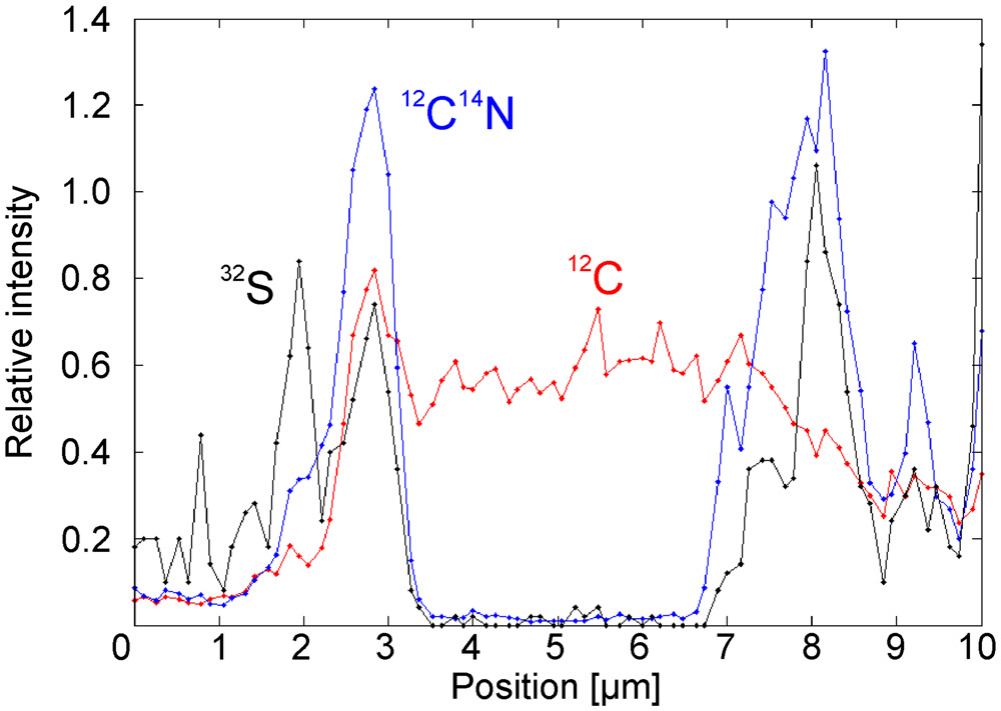
NanoSIMS isotope enrichment profile across a spheroid (see Fig 8 for orientation of the section). Organic matter (^12^C^14^N) and sulfur (^32^S) are closely associated and preferentially enriched at the edges of the body, whereas carbon (^12^C) is also enriched in intermediate spaces due to the presence of carbonate phases.

NanoSIMS isotope maps of organic layers shown in Fig 7A reveal the presence of clusters of spheroidal bodies with sizes up to several tens of μm that are enriched in C compared to the surrounding areas (Fig 8). S and N are preferentially enriched at the edges but strongly depleted in the centers of these bodies (Figs 8 and 9).

The bulk δ^13^C value of organic matter in the black chert facies is -35.3‰. Framboidal pyrite crystals exhibit a δ^34^S value of +3.05‰, and Δ^33^S and Δ^36^S values of 0.268‰ and -0.282‰, respectively.

## Discussion

### Sedimentary facies and paleoenvironment

The black chert facies contains layers of cm-high domical stromatolites, underlining the general habitability of the paleoenvironment. These stromatolites exhibit many characteristic features of more recent stromatolites at the micro-scale, including steep-sided growth walls that truncate laminae, internal discordances of laminae, and growth termination by influx of clastic sediment [6], [7]. The cross laminated sandstones of the lower part of the facies (Fig 3E), stromatolitic layers that show ductile deformation (Fig 3D) and crescentic, wind-blown ripples formed nearby [7] indicate deposition under extremely shallow water conditions.

**Fig 10:**
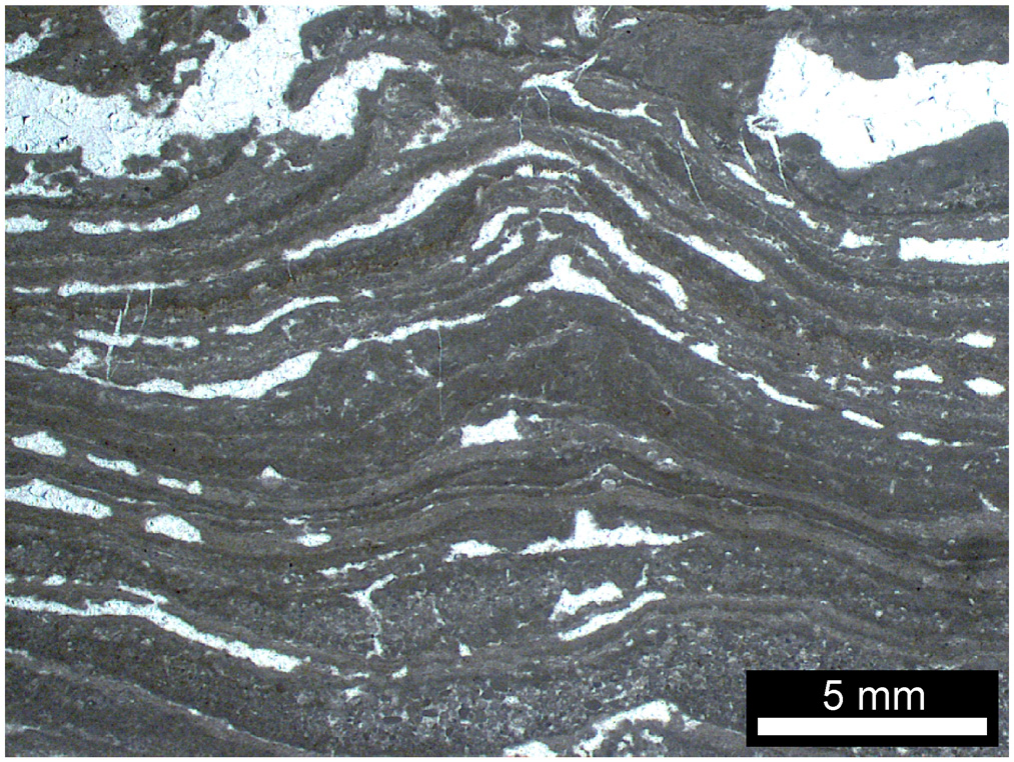
Fenestral carbonate facies from the Triassic Dachstein Limestone of the Northern Calcareous Alps. This facies was formed by microbial mats in peritidal- to shallow lagoonal environments.

Multiple cementation stages in the white quartz layers and lenses (Fig 4B and 4D) of the black chert facies most likely originate from open cavities (i.e. fenestrae) that were filled by carbonate and quartz cements. Similar cementation patterns are known from microbial mats in the 3.42 Ga Buck Reef Chert in South Africa (e.g., [52]–[54]) and from numerous Phanerozoic peritidal- to shallow lagoonal microbial carbonates (e.g., the Triassic Dachstein Limestone of the Northern Calcareous Alps, [55], [56]; Fig 10). Fenestrae have been interpreted to result from the decay of microbial mat-derived organic matter and subsequent compaction of gas bubbles (e.g., [55]–[58]). Alternatively, fenestrae may have resulted from patches of incompletely degraded microbial exopolymers that locally inhibited mineral precipitation. The nucleation-inhibiting nature of exopolymers is well known from recent microbial mats (e.g. cf. [59]). In any case, the features observed in the black chert facies are fully consistent with a microbial mat environment. The acicular (i.e. needle-shaped) crystals with pseudo-hexagonal cross-sections may represent chert pseudomorphs after aragonite (Fig 4G and 4H), as these habits are typical features of modern aragonite cements (e.g., [56], [60], [61]). Given also the rhombohedral dolomite observed in some of the fenestrae (Fig 6C and 6D), our results do not point to a primary hydrothermal setting [26], [62]. Our findings support previous interpretations that the Strelley Pool Formation was deposited in a shallow marine microbial mat environment, and that carbonate precipitation and silicification occurred during sedimentation and earliest diagenesis prior to the deposition of the overlying Member IV (e.g., [5], [7]-[9], [11], [18]-[20], [22], [35]).

### Geomicrobiological implications

The inferred shallow water setting of the black chert facies would have provided a suitable habitat for photoautotrophs within the microbial mats. Such a scenario is consistent with the δ^13^C_Org_ values observed (-35.3‰, this study; -35.2‰, [63]), which are in the range of biomass from extant photoautotrophs (down to c. -35‰; cf. [64]).

A further important trait of the black chert facies is the close association of organic material and framboidal pyrite in distinct layers (Fig 4C, 4E and 4F). Although such crystals can also be formed abiotically (see [65] for a review), sedimentary pyrite framboids mainly result from microbial sulfate reduction (e.g., [66], [67]). In fact, the close association between framboidal pyrite and organic material is a typical feature of fossil and recent biofilms (e.g., [68]-[70]). Therefore, the relatively high abundance of framboidal pyrite is interpreted to result from microbial sulfur turnover within the microbial mat.

Archean sedimentary sulfur is typically characterized by non-zero Δ^33^S and Δ^36^S values (see [71] for a recent review) resulting from UV-induced processing of volcanogenic SO_2_ within an anoxic atmosphere (pO_2_ <10^−5^ of the present day atmospheric level of oxygen, cf. [72]). Attenuated non-zero Δ^33^S and Δ^36^S values in the black chert facies (0.268‰ and -0.282‰, respectively) would be consistent with such a photolytic source of the pyrite sulfur.

Similar to microbial sulfate reduction, the disproportionation of elemental sulfur or thiosulfate typically results in a sizeable mass-dependent fractionation of ^32^S and ^34^S (expressed as δ^34^S; see [73]). Unfortunately, the δ^34^S signature in the black chert facies (+3.05‰) provides no clue to microbial sulfur processing because the isotopic composition of putative substrates in the Strelley Pool Formation is unknown. Nevertheless, the sulfur isotope data are consistent with a microbial origin of the framboidal pyrite and the proposed presence of microbial sulfur metabolism elsewhere in the Strelley Pool Formation [13], [15], [16], [74].

### Taphonomy and origin of the spheroidal bodies

Compared to the Triassic Dachstein Limestone of the Northern Calcareous Alps, the black chert facies appears to be much less compacted (e.g. fenestrae in Figs 4A, 4B, 4D and 10). This is fully consistent with synsedimentary to very early diagenetic silicification of the Strelley Pool Formation due to hydrothermal inputs [7], [11], [22], [36]. Comparable processes are known from the Rhynie Chert (Devonian, NE Scotland), where fragile plant remains are locally preserved in growth position and lack evidence of decay (e.g., [75]–[77]). This exceptional preservation is commonly three-dimensional due to a combination of silica coating on organic surfaces and permineralization (i.e. mineral in-fills of inter- and intracellular cavities, but preservation of primary organic tissue) (e.g., [75]-[77]). Synsedimentary- to very early diagenetic silification could thus account for the preserved organic materials (Figs 4B, 4C, 5, 6A and 6B), the framboidal pyrite (Fig 4E and 4F), the carbonates (Figs 5, 6C, 6D and 7) and chert pseudomorphs after possible aragonite (Fig 4G and 4H). Furthermore, it is in good accordance with the preservation of carbonaceous fossils in other black cherts of the Strelley Pool Formation that also exhibit fenestral structures [9], [11].

The spheroidal bodies described here (Fig 8) are interpreted as being authigenic as they were observed in a pristine inner part of a sample, which lacks cracks or fissures. Interestingly, they resemble small (<15 μm) carbonaceous spheroids in other black cherts of the Strelley Pool Formation that commonly form colony-like clusters [9], [10]. Furthermore, globular bodies enriched in C, N and S have been observed in the basal sandstone member of the Strelley Pool Formation [78] as well as in strata of different ages such as the Farrel Quarzite (3000 Ma, Western Australia) and the Bitter Springs Formation (c. 850 Ma, Northern Territory, Australia) [79]–[81]. In all these cases, morphology and biogeochemical signatures were taken as evidence for a biogenic origin, and we interpret the spheroidal bodies investigated herein accordingly.

The C-enriched central parts of the spheroids are extremely lean in S and N (Figs 8 and 9) and are thus unlikely to represent organic matter. Instead, small carbonate grains have been detected by CL microscopy (Fig 7), and a close spatial association between carbonate and organic matter within the chert matrix has been revealed by ToF-SIMS (Fig 5). Therefore, the central parts of the spheroids are interpreted as carbonate minerals that are surrounded by biogenic organic material and pyrite.

The observed close spatial association of biomass and carbonate could be due to active intracellular carbonate precipitation by bacteria. This process is generally rare but known from phylogenetically old unicellular cyanobacteria of the order Gloeobacterales (e.g., [82], [83]) and from the sulfur bacterium *Achromatium oxaliferum* (e.g., [84], [85]). However, the active intracellular formation of carbonate is enzymatically complex, and it is questionable if the required enzymes already existed in the early Archean. A second possibility for the observed features is the degradation of organic matter by heterotrophic microbes that caused a local change of geochemical microenvironment and led to the precipitation of carbonates (cf. [86]). Likewise, the preferential formation of Mn^2+^-enriched carbonates, as indicated by the strong CL (Fig 7), would be in line with a microbial impact. In fact, microbially induced rhodochrosite formation is known from a number of bacteria and archaea (e.g., [87]–[89]).

## Conclusions

The black chert facies with fenestral fabrics from the Trendall locality of the 3.4 Ga old Strelley Pool Formation is interpreted as a silicified microbial mat, deposited in a shallow water environment. The biogenicity of this facies is indicated by the similarity to microbial carbonate facies of younger age, the presence of stromatolites, and the close association between organic matter and framboidal pyrite. Carbonate precipitation and silicification by hydrothermal fluids occurred during sedimentation and earliest diagenesis. The silicification accounts for the non-compacted nature of the facies and the exceptional preservation of the organic matter, framboidal pyrite, and carbonates. Particularly important are spheroidal carbonate bodies that are surrounded by organic material and pyrite. These spheroids are tens of μm in diameter and are interpreted as biogenic. Although the metabolisms of the inferred microbes remain to be resolved, our data are consistent with the existence of phototrophic and sulfur-based pathways in the Paleoarchean mat system.
